# Evaluation of the CalGrows Innovation Fund Direct Care Training and Stipends Program

**DOI:** 10.1093/geroni/igaf122.1834

**Published:** 2025-12-31

**Authors:** Jacqueline Miller, Timothy Bates, Xenia Mendez, Joanne Spetz

**Affiliations:** University of California, San Francisco, San Francisco, California, United States; University of California, San Francisco, San Francisco, California, United States; University of California, San Francisco, San Francisco, California, United States; University of California, San Francisco, San Francisco, California, United States

## Abstract

**Research Objective:**

The California Growing a Resilient and Outstanding Workforce (CalGrows) program distributed $150 million in grant funding to provide free training to direct care workers (DCWs). DCWs could earn up to $7,000 in training stipends. This study evaluated the design and delivery of CalGrows training courses and DCWs’ and caregivers’ attitudes about their training experiences.

**Study Design:**

A survey measuring DCWs’ and caregivers’ perceptions of their CalGrows training experience was sent to training participants. Interviews were conducted with training providers, DCWs, and program administrators to understand program design and delivery, perceptions of training’s impact on confidence in skills, and quality of care.

**Principal Findings:**

CalGrows funded 76 organizations with about 850 different training courses offered in varying formats. More than 18,500 DCWs completed at least one training course. Approximately 90% of participants indicated that the training made them feel better prepared across a range of knowledge and skill areas. Over 90% agreed that the training made them want to remain in their current job or the caregiving field. More than 95% felt that the CalGrows training improved the quality of care they provide and their confidence. Grantees who provided training to their own employees described dramatic improvements in employee retention. Training providers emphasized the importance of promoting their training through a trusted network in the home and community-based services sector and the need for sustainable funding.

**Conclusions:**

The CalGrows program demonstrated the need for and value of providing free and accessible training to DCWs.

